# Initial Characterization of Stressed Transgenic Mice With Cardiomyocyte-Specific Overexpression of Protein Phosphatase 2C

**DOI:** 10.3389/fphar.2020.591773

**Published:** 2021-01-11

**Authors:** Paula Bollmann, Franziska Werner, Marko Jaron, Tom A. Bruns, Hartmut Wache, Jochen Runte, Peter Boknik, Uwe Kirchhefer, Frank U. Müller, Igor B. Buchwalow, Sven Rothemund, Joachim Neumann, Ulrich Gergs

**Affiliations:** ^1^ Institut für Pharmakologie und Toxikologie, Medizinische Fakultät, Martin-Luther-Universität Halle-Wittenberg, Halle, Germany; ^2^ Institut für Pharmakologie und Toxikologie, Medizinische Fakultät, Westfälische Wilhelms-Universität, Münster, Germany; ^3^ Institute for Hematopathology, Hamburg, Germany; ^4^ IZKF Leipzig, Leipzig, Germany

**Keywords:** transgenic mice, PP2A, PP2C, heart failure, fibrosis, inflammation

## Abstract

As part of our ongoing studies on the potential pathophysiological role of serine/threonine phosphatases (PP) in the mammalian heart, we have generated mice with cardiac-specific overexpression of PP2Cβ (PP2C-TG) and compared them with littermate wild type mice (WT) serving as a control. Cardiac fibrosis was noted histologically in PP2C-TG. Collagen 1a, interleukin-6 and the natriuretic peptides ANP and BNP were augmented in PP2C-TG vs. WT (*p* < 0.05). Left atrial preparations from PP2C-TG were less resistant to hypoxia than atria from WT. PP2C-TG maintained cardiac function after the injection of lipopolysaccharide (LPS, a model of sepsis) and chronic isoproterenol treatment (a model of heart failure) better than WT. Crossbreeding of PP2C-TG mice with PP2A-TG mice (a genetic model of heart failure) resulted in double transgenic (DT) mice that exhibited a pronounced increase of heart weight in contrast to the mild hypertrophy noted in the mono-transgenic mice. The ejection fraction was reduced in PP2C-TG and in PP2A-TG mice compared with WT, but the reduction was the highest in DT compared with WT. PP2A enzyme activity was enhanced in PP2A-TG and DT mice compared with WT and PP2C-TG mice. In summary, cardiac overexpression of PP2Cβ and co-overexpression of both the catalytic subunit of PP2A and PP2Cβ were detrimental to cardiac function. PP2Cβ overexpression made cardiac preparations less resistant to hypoxia than WT, leading to fibrosis, but PP2Cβ overexpression led to better adaptation to some stressors, such as LPS or chronic β-adrenergic stimulation. Hence, the effect of PP2Cβ is context sensitive.

## Introduction

Serine and threonine phosphatases (PP) such as PP1, PP2A, PP2B, PPC, PP4 and PP5 are present in the cardiomyocytes of mice and humans (Herzig and Neumann, 2000; DeGrande et al., 2013; Gergs et al., 2019a). PP activity is enhanced in some forms of human heart failure and other cardiac diseases such as arrhythmias; some of these pathologies could be recapitulated in animal models of hypertrophy, failure and cardiac arrhythmias (Neumann et al., 1997; Bokník et al., 2000; for a review see: Dobrev et al., 2019; Dobrev and Wehrens, 2018). Vice versa, mice with an overexpression of catalytic subunits of PP, for example, PP1, PP2A, PP2B, PP2C or PP5, exhibited various degrees of hypertrophy, heart failure and arrhythmias (Molkentin et al., 1998; Gergs et al., 2004; Brüchert et al., 2008; Gergs et al., 2012) but exhibited increased stress resistance under certain experimental conditions (Morita et al., 2001; Hoehn et al., 2015; Neumann et al., 2019). The phenotype of these mice encompasses reduced phosphorylation of cardiac regulatory proteins in various subcellular compartments of cardiomyocytes. The regulatory proteins of interest in this context reside in the sarcolemma, the sarcoplasmic reticulum, the mitochondria and the nucleus of cardiac cells (Herzig and Neumann, 2000). The dephosphorylations of these regulatory proteins are thought to explain (at least in part) the phenotypes of these as a type of impaired force generation and a prolonged relaxation of developed force; similar observations have been made in failing human hearts (Bartel et al., 1996; Herzig and Neumann, 2000). Based on these data, inhibitions of the enzymatic activity of some phosphatases with small organic molecules (tacrolimus for PP2B, okadaic acid for PP1: Neumann et al., 1993; Neumann et al., 1994; Neumann et al., 1995; Herzig and Neumann, 2000) or endogenous proteins such as inhibitor-1 and inhibitor-2 (El-Armouche et al., 2004; Kirchhefer et al., 2005; Pathak et al., 2005; Brüchert et al., 2008; Grote-Wessels et al., 2008; Kirchhefer et al., 2018; Krause et al., 2018) have been suggested to be of potential benefit in heart failure patients (Pathak et al., 2005).

Whereas about 400 different kinases increase the phosphorylation state of regulatory proteins, fewer PP are known (about 30: for a review, see Brautigan and Shenolikar, 2018). Although protein kinases gain specificity because of the motifs of the amino acids around potentially phosphorylated serines or threonines, the evolution of PPs has put forth another approach. Initially, the hypothesis was that PP are passive and just reverse the action of kinases without any regulation of their function. Later, it became clearer that at least PP1 can regulate its activity through more than 40 additional regulatory proteins, including the so-called inhibitors I-1 and I-2. Likewise, it is becoming clearer that the activity of the other main cardiac PP, PP2A, is regulated by ancillary proteins (DeGrande et al., 2013).

Very little is known about PP2C in the heart. It is known that PP2C is present in the human heart as demonstrated on mRNA level (Marley et al., 1998) and on protein level by immunohistology in human ventricular cardiomyocytes (Lifschitz-Mercer et al., 2001) or by Western blotting in human atrium (Gergs et al., 2019a). The cloning, expression and tissue distribution of human PP2Cβ was described, however, quite early on (Marley et al., 1998). The PP2C family forms part of the PPM family of metal ion-dependent PP (review: Grzechnik and Newton, 2016). Interestingly, PP2C is present in cardiac mitochondria and might regulate their function and also their density in skeletal muscle cells suggesting a disease-specific role. This was demonstrated in a mouse model by siRNA-mediated knockdown of PP2C leading to inhibition of angiotensin II-induced mitochondrial dysfunction (Tabony et al., 2014).

These PPMs have catalytic subunits, but their regulation is only slowly being understood. Binding proteins like for PP1 and PP2A have not been uncovered for PPMs. However, some regulation of PPMs exists because, for instance, PPM activity is enhanced by Mg^2+^. Physiologically, Mg^2+^ concentrations (e.g., in the rat heart, Mg^2+^ was measured as 0.85 mM: Murphy et al., 1989) can stimulate PP2C activity. Moreover, PP2C is activated by fatty acids such as oleic acid. Moreover, oleic acid can impair the morphology of neonatal mouse cardiomyocytes in culture, leading to apoptosis; this apoptosis was reduced after a knockdown of PP2Cβ that was introduced using antisense RNA against PP2Cβ (Krieglstein et al., 2010).

PPM family members occur early in evolution; for example, CYR1 is a PP2C homologue in yeast (review: Grzechnik and Newton, 2016). A knockout of CYR1 in yeast is lethal (review: Grzechnik and Newton, 2016), and likewise, a knockout of PP2Cβ is embryonally lethal in mice (Sasaki et al., 2007). Members of the PPM family like PHLPP have been identified as tumor suppressors (Grzechnik and Newton, 2016), which might suggest disease-related function of PP2C in human diseases. A well-known substrate of PP2C is Akt, which is known to ensure survival of cardiomyocytes in the heart (Grzechnik and Newton, 2016). Hence, because many substrates for PP2C are present in the mammalian heart, it is plausible that PP2C could play a role in cardiac diseases such as heart failure and arrhythmias, but data to support this hypothesis are absent. This led us to initiate the present project.

In the present work, the effects of the overexpression of PP2Cβ and, for comparison, the catalytic subunit of PP2A on cardiac performance were studied. Moreover, we stressed PP2C-TG by sepsis, acute and chronic β-adrenergic stimulation and hypoxia. Parts of the results have been published in abstract form (Bollmann et al., 2015; Runte et al., 2017; Bruns et al., 2018; Jaron et al., 2018; Bollmann et al., 2019; Bollmann et al., 2020).

## Materials and Methods

### Transgenic Mice

**FIGURE 1:**
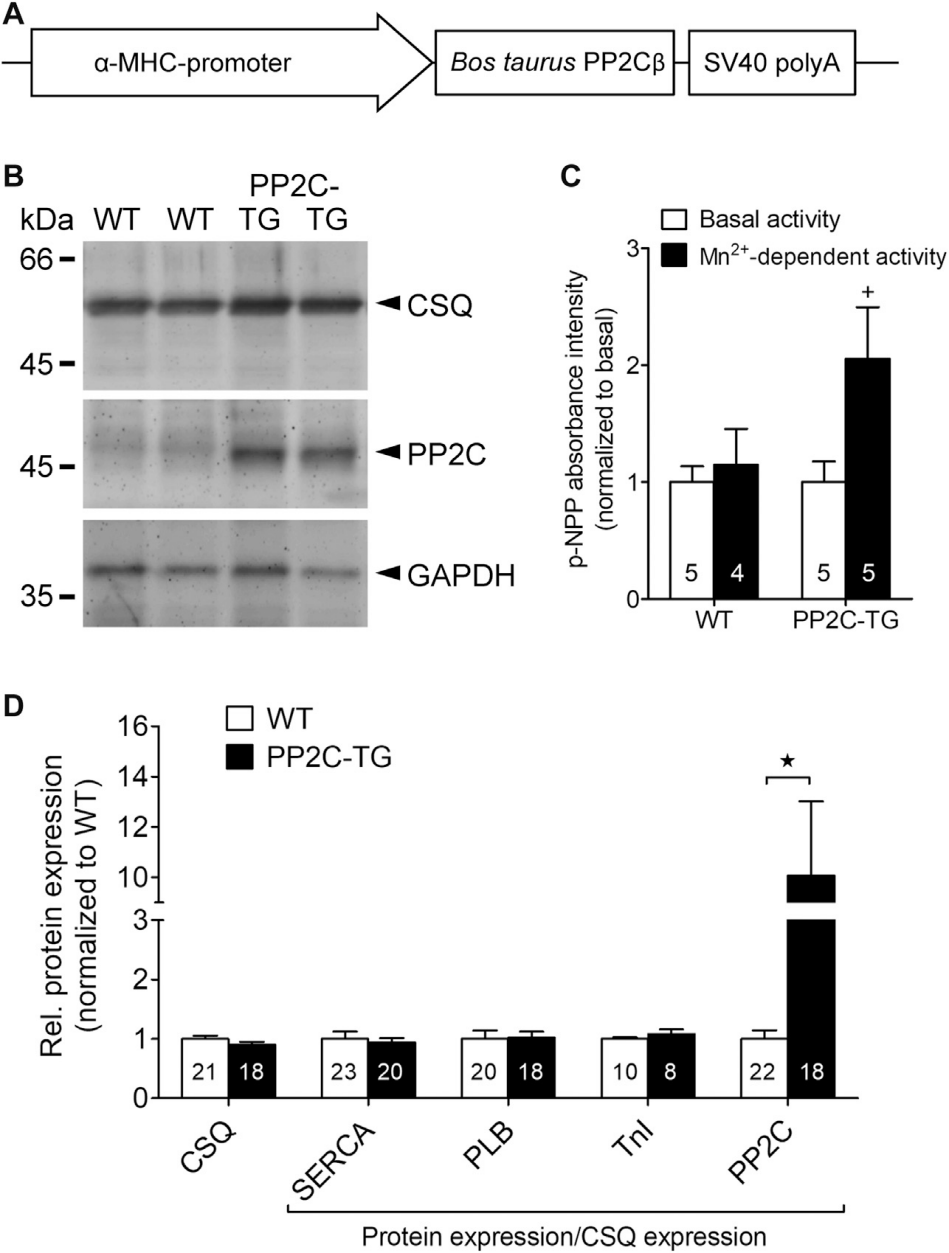
Generation of transgenic mice. **(A)** Transgenic construct: the cDNA of bovine PP2Cβ was overexpressed under the control of the α-myosin heavy chain (α-MHC) promoter, which conveys cardiac myocyte selectivity of gene expression. **(B)** Western blot experiments confirmed a successful overexpression of PP2Cβ protein in PP2C-TG hearts (TG) compared to WT. Molecular weight standards are indicted in kilo Daltons (kDa) on the left hand side of the gel. Cardiac calsequestrin (CSQ) and glyceraldehyde-3-phosphate dehydrogenase (GAPDH) detection on the blotting membrane indicates equal protein loading. **(C)** The putative activity of PP2C in cardiac homogenates was determined using the artificial substrate p-nitrophenyl phosphate (p-NPP) in presence of Mn^2+^. **(D)** Quantitative analysis of Western blot experiments revealed unchanged cardiac protein expression of CSQ, sarco/endoplasmic reticulum Ca^2+^ ATPase (SERCA), phospholamban (PLB), and troponin inhibitor (TnI) in PP2C-TG hearts compared to WT. Moreover, PP2C was nearly tenfold overexpressed in PP2C-TG hearts. ★*p* < 0.05 vs. WT; ^+^ *p* < 0.05 vs. basal.

Transgenic mice with cardiomyocyte-specific overexpression of the catalytic subunit of PP2A (PP2Acα) were generated utilizing a mouse cardiac α-myosin heavy chain promoter expression cassette containing the cDNA of mouse PP2Acα along with 326 base pairs of the 3’ untranslated region and 69 base pairs of the 5’ untranslated region, as described previously (Gergs et al., 2004). In a similar fashion, PP2C overexpression mice were generated for the present study (Figure 1). The cDNA for bovine PP2Cβ (GenBank: AJ005458.1) was provided by S. Klumpp (Münster, Germany). All mice were housed under conditions of optimum light, temperature and humidity, with food and water provided ad libitum. The animals were handled and maintained according to approved protocols of the animal welfare committee of the University of Halle-Wittenberg, Halle, Germany.

### Contractile Studies in Mice

To address the atrial function *in vitro*, right or left atrial preparations were isolated and mounted in organ baths, as described before (Neumann et al., 2003; Gergs et al., 2013). The bathing solution of the organ baths contained (in mM) NaCI, 119.8; KCI, 5.4; CaCl_2_ 1.8; MgCl_2_, 1.05; NaH_2_PO_4_, 0.42; NaHCO_3_, 22.6; Na_2_EDTA, 0.05; ascorbic acid, 0.28; and glucose, 5.05; the bath was continuously gassed with 95% O_2_ and 5% CO_2_ and maintained at 37°C and pH 7.4, as described previously (Neumann et al., 2003; Kirchhefer et al., 2004). Preparations were attached to a bipolar stimulating electrode and suspended individually in 10 ml glass tissue chambers for recording isometric contractions. The force of the contraction was measured with inductive force transducers connected to a digitiser. Contractile parameters were analyzed using Labchart 8 (ADInstruments, Oxford, United Kingdom). Each muscle was stretched to the length of the maximal force of contraction. The left atrial preparations from mice and the human preparations were electrically stimulated at 1 Hz with rectangular pulses of 5 ms duration; the voltage was ∼10–20% greater than the threshold. The contractile parameters were evaluated. The force of contraction was the difference between the maximum and minimum tension at constant muscle length. Spontaneously beating right atrial preparations from the mice were used to study any chronotropic effects.

To address the ventricular function *in vitro*, Langendorff perfusion experiments were performed as published before with slight modifications (Gergs et al., 2019b). In brief, spontaneously beating hearts were retrogradely perfused under constant flow (2 ml/min) and the force of contraction was measured mechanically at the apex of the heart. After 15 min of equilibration, hypoxia was induced for 20 min by stop of the perfusion followed by reperfusion for 15 min. Finally, 1 µM isoproterenol was applied. Time controls were perfused for 50 min under normoxic conditions and finally stimulated with 1 µM isoproterenol. Before stop of perfusion (basal parameters), at the end of stop of perfusion, after reperfusion (hypoxia parameters), and at the maximum effect of isoproterenol force parameters were assayed.

### Echocardiography

Transthoracic echocardiographic measurements in spontaneously breathing mice were performed under anesthesia with 1.5% isoflurane using a Vevo 2,100 system equipped with a MS 550D transducer (Visual Sonics, Toronto, Canada). Two-dimensional images and M-mode tracings were recorded from the parasternal long axis view. The cardiac dimensions were measured, and the ejection fraction of the hearts was calculated. In addition, the Doppler option of Vevo 2,100 was used for arterial and venous flow measurements, as described previously (Gergs et al., 2004; Gergs et al., 2010; Gergs et al., 2019b).

### Expression of PP2C and Dephosphorylation of Substrates

PP2Cβ (bovine cDNA made available from S. Klumpp, Münster, Germany; Klumpp et al., 1998) was expressed in *E. coli* and purified by column chromatography following published procedures; it was found to be stimulable with 0.7 mM MgCl_2_ using ^32^P-casein (McGowan and Cohen, 1988) as a substrate. Membranes (Frank and Overwin, 1996) that contained sequences of described phosphorylatable motifs of common cardiac phosphoproteins, based on a similar published protocol (Neumann et al., 2007; Werner et al., 2007), were prepared. These membranes were phosphorylated by incubation with a catalytic subunit of protein kinase A and _ϒ_^32^P-ATP. Phosphorylation of the spots was confirmed by autoradiography. The spots were dephosphorylated by incubation with PP2C. Dephosphorylation was likewise confirmed by autoradiography.

### Western Blot Analysis

For the Western blot analysis, ventricular homogenates were prepared, and aliquots of 50–200 μg protein were loaded per lane, as described previously (Gergs et al., 2004). Protein loading was monitored by Ponceau staining of the nitrocellulose membranes and expression of calsequestrin (CSQ). CSQ was used for normalization of protein expression because it’s a marker of cardiac myocytes. Bands were detected using enhanced chemifluorescence (ECF) and a Typhoon 9,410 Variable Mode Imager (GE Healthcare, Freiburg, Germany). The signals were quantified with the ImageQuant TL software (GE Healthcare, Freiburg, Germany). The list of primary antibodies used is summarized in the section titled ‘Drugs and Materials’. Corresponding secondary antibodies conjugated with alkaline phosphatase were purchased from Sigma-Aldrich (Munich, Germany).

### Protein Phosphatase Assay PP2A, PP1

Phosphorylase phosphatase activity (for PP1 and PP2A) was determined, as described previously (Neumann et al., 1993), with [^32^P]-phosphorylase as the substrate. Portions (20 mg) of pulverized frozen tissue were homogenized at 4°C three times for 30 s each with a Polytron PT-10 (Kinematica, Luzern, Switzerland) in a 300 µl buffer containing (in mmol/L) EDTA 4, β-mercaptoethanol 15, pH 7.4. The homogenate was centrifuged for 20 min at 14,000 g. The incubation mixture contained (mmol/L) TRIS HCl (pH 7.0) 20.0, caffeine 5.0, EDTA 0.1 and β-mercaptoethanol 0.1% (vol/vol). The reaction was started by adding aliquots of homogenates (containing 3–11 µg protein) or aliquots of peak fractions. The samples were assayed in the presence and absence of 3 nM okadaic acid, which completely inhibits PP2A activity; thus, the remaining activity would only occur because of PP1 (as described in Kirchhefer et al., 2014). The reaction was stopped by the addition of 50% trichloroacetic acid. The precipitated protein was sedimented by centrifugation, and the radioactivity in the supernatant was counted in a liquid scintillation counter.

### Phosphatase Assay PP2C

The activity of PP2C in cardiac homogenates was determined using the artificial substrate p-nitrophenyl phosphate (pNPP) in presence of Mn^2+^ according to the modified protocol of Su and Forchhammer (2013). Briefly, portions (20 mg) of frozen tissue were homogenized in 500 µl buffer (10 mM Tris/HCl, 50 mM NaCl, 1 mM dithiothreitol, pH 8.0) under liquid N_2_ in a Mikro-Dismembrator ball mill (1 min, 2,700 rpm). After thawing, the homogenates were cleared by centrifugation at 4°C (10 min, 15,000x g). Each sample was measured as triplicate either in presence of 2 mM MnCl_2_ or as control for basal activity without Mn^2+^. Reactions were started by the addition of 20 mM p-NPP at 37°C. After 10 min, reaction was stopped by 100 mM K_2_HPO_4_, pH 10 and the absorbance at 405 nm was measured. For background absorbance, a heat inactivated sample (10 min at 95°C) was used.

### Repeated Isoproterenol Treatment

As a heart failure model, 0.1 mg/g body weight of isoproterenol bitartrate (diluted in isotonic sodium chloride solution) was once daily injected intraperitoneally over four consecutive days. Control injections contained only isotonic sodium chloride solution (Brooks and Conrad, 2009; Ma et al., 2018).

### LPS Treatment

The mice were treated with intraperitoneal injection of 30 µg/g body weight LPS (055 B5, Sigma) diluted in isotonic sodium chloride solution. The control injections contained only isotonic sodium chloride solution (Gergs et al., 2019c).

### Histological Analysis

Histological assessment was performed as described before (Gergs et al., 2010; Gergs et al., 2019b).

### Real Time Polymerase Chain Reaction (qPCR)

The total RNA was isolated using the TRIzol reagent (Invitrogen, Fisher Scientific, Schwerte, Germany) according to the manufacturer’s instructions. Subsequently, reverse transcription was performed using the Maxima First Strand cDNA Synthesis Kit (Fisher Scientific, Schwerte, Germany) according to the manufacturer’s instructions. During cDNA synthesis, the remaining DNA was digested with DNase I. Reverse transcription was performed with 2–5 μg RNA and a mixture of oligo (dT)18 and random hexamer primers. As a control, each RNA sample was also analyzed without reverse transcription (NRT). Finally, cDNA samples were diluted to a volume corresponding to 0.005 μg RNA per μl. Real-time PCR amplification and detection was performed with the BioRad CFX Connect system using the iTaq SYBR Green kit (BioRad Laboratories, Munich, Germany) according to the manufacturer’s instructions. The relative expression of the genes of interest was calculated according to the 2^−ΔΔCT^ method (Livak and Schmittgen, 2001) by using the GAPDH signal for normalization. The primers were either developed with the software Discovery Studio Gene v1.5 (Accelrys, Cambridge, United Kingdom) or were derived from the literature (Wu et al., 2002; Chen et al., 2006; Yamamoto et al., 2009; Hajjar et al., 2012; Furlow et al., 2013). The list of primer sequences used is summarized in the section titled ‘Drugs and Materials’.

### Data Analysis

The data shown are means ± SEM. Statistical significance was estimated by an analysis of variance (ANOVA) followed by Bonferroni’s t-test or by using the student’s t-test when appropriate. A *p*-value < 0.05 was considered significant. Experimental data for agonist-induced positive inotropic and chronotropic effects were analyzed by fitting the sigmoidal curves to the experimental data using GraphPad Prism 5.0. All other statistical analyses were performed as indicated in the figures and tables. A statistical evaluation was conducted with GraphPad Prism 5.0 (GraphPad Software, San Diego, California, United States), which was also used to produce graphs.

### Drugs and Materials

The peptide sequences used were as follows (the putative phosphorylation sites are numbered):

L-type Ca^2+^-channel: LTCCa1 S1487P: NFDYLTRDWSILGPHHLDE
C-protein: MBP-C S282P: SLAGAGRRTSDSHEDAGTP
Troponin inhibitor S23P: PAPAPVRRRSSANYRAYAT
Phospholamban PLB S16P: LTRSAIRRASTIEMPQQAR
β_2_-adrenoceptor: B2AR S346P: QELLCLRRSSSKTYGNGYS
Phosphodiesterase PDE4D3 S129P: NFVHSQRRESFLYRSDSDY
Phosphodiesterase PDE3B Ser10 GIPEMFRRPSLPCISREQM

(−)-Isoproterenol (+)-bitartrate was purchased from Sigma-Aldrich (Deisenhofen, Germany). All other chemicals were of the highest purity grade commercially available. Deionized water was used throughout the experiments. Stock solutions were freshly prepared daily.

Antibodies: anti PP2C (PPM1B), proteintech #13193-1-AP (1:500); anti calsequestrin (CSQ), abcam #ab3516 (1:1,000); anti GAPDH, abcam #mAbcam9484 (1:1,000); anti SERCA, kindly provided by L.R. Jones, Indianapolis, IN, United States (1:1,000); anti phospholamban, Badrilla #A010-14 (1:2,000); anti troponin inhibitor (TnI), Cell Signaling #13083 (1:1,000).

Primer sequences: ANP, forward, GTG​CGG​TGC​CAA​CAC​AGA​T, reverse, GCT​TCC​TCA​GTC​TGC​TCA​CTC​A; BNP, forward, CCA​GTC​TCC​AGA​GCA​ATT​CAA, reverse, AGC​TGT​CTC​TGG​GCC​ATT​TC; Col1a1, forward, ACA​TGT​TCA​GCT​TTG​TGG​ACC, reverse, TAG​GCC​ATT​GTG​TGT​ATG​CAG​C; Col3a1, forward, TGG​TAG​AAA​GGA​CAC​AGA​GGC, reverse, TCC​AAC​TTC​ACC​CTT​AGC​ACC; Fn1, forward, TTA​AGC​TCA​CAT​GCC​AGT​GC, reverse, TCG​TCA​TAG​CAC​GTT​GCT​TC; GAPDH, forward, ATG​CAT​CCT​GCA​CCA​CCA​AC, reverse, ATG​CCT​GCT​TCA​CCA​CCT​TC; IL-1b, forward, TCG​TGC​TGT​CGG​ACC​CAT​AT, reverse, GTC​GTT​GCT​TGG​TTC​TCC​TTG​T; IL-6, forward, CCG​GAG​AGG​AGA​CTT​CAC​AG, reverse, TTC​TGC​AAG​TGC​ATC​ATC​GT; IkBa, forward, ATG​AAG​GAC​GAG​GAG​TAC​GAG​CAA, reverse, TCT​CTT​CGT​GGA​TGA​TTG​CCA​A; NFkB, forward, GAA​ATT​CCT​GAT​CCA​GAC​AAA​AAC, reverse, ATC​ACT​TCA​ATG​GCC​TCT​GTG​TAG; TNFa, forward, CAC​ACT​CAG​ATC​ATC​TTC​TCA​AAA, reverse, GTA​GAC​AAG​GTA​CAA​CCC​ATC​G.

## Results

**FIGURE 2:**
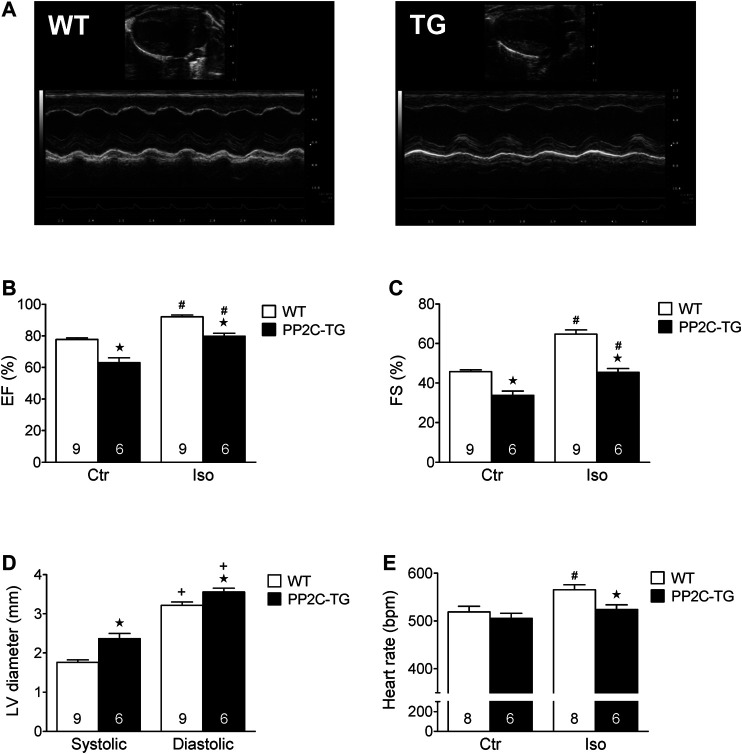
*In vivo* cardiac function of PP2C-transgenic (PP2C-TG) mice using echocardiography. **(A)** Original B-mode and M-mode images. **(B)** We noted a reduced ejection fraction (EF) in PP2C-TG compared to wild type (WT) mice under control conditions (Ctr) (WT 78 ± 1%; TG 63 ± 3%) as well as after intraperitoneal application of isoproterenol (Iso) (WT 92 ± 1%; PP2C-TG 80 ± 2%). **(C)** Fractional shortening (FS) was also reduced in PP2C-TG under control conditions and after isoproterenol injection (from 46 ± 1 to 34 ± 2% in WT and from 65 ± 2 to 45 ± 2% in PP2C-TG). **(D)** We measured higher diastolic and systolic left ventricle (LV) diameters in PP2C-TG mice (systolic: WT 1.76 ± 0.06 mm, PP2C-TG 2.37 ± 0.13 mm; diastolic: WT 3.22 ± 0.08 mm, PP2C-TG 3.56 ± 0.1 mm). **(E)** Compared to WT, the isoproterenol-induced increase in heart rate (in beats per minute, bpm) was diminished in PP2C-TG mice (from 519 ± 12 bpm to 566 ± 9 bpm in WT and from 505 ± 11 bpm to 524 ± 10 bpm in PP2C-TG). ★*p* < 0.05 vs. WT; ^#^ *p* < 0.05 vs. Ctr; ^+^ *p* < 0.05 vs. systolic.

**FIGURE 3:**
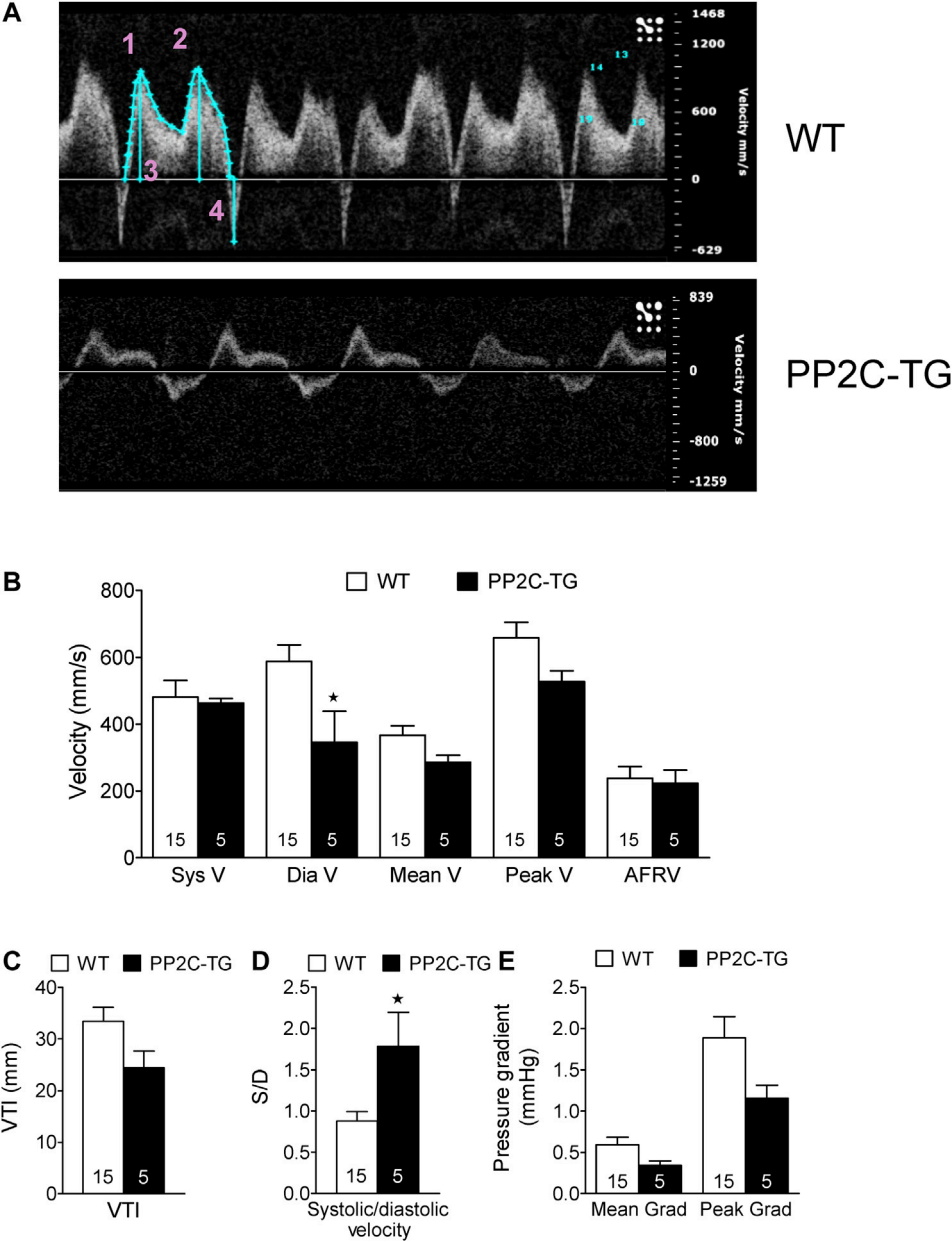
Pulsed wave Doppler echocardiography. **(A)** Vena pulmonalis, original recordings. 1: Systolic velocity (S wave), 2: Diastolic velocity (D wave), 3: Volume time integral, 4: Atrial flow reversal velocity (AFRV). **(B)** Velocities in mm/s, **(C)** Velocity time integral (VTI) in mm, **(D)** S/D ratio (dimensionless), **(E)** Mean and peak gradient (Mean Grad, Peak Grad) in mmHg. Whereas the systolic and atrial flow reversal velocity remained unchanged between WT and PP2C-TG, the diastolic velocity decreased in PP2C-TG and the resulting S/D ratio was elevated. The flow parameters VTI, mean velocity, mean gradient, peak velocity and peak gradient did not differ between WT and PP2C-TG. ★*p* < 0.05 vs. WT.

**FIGURE 4:**
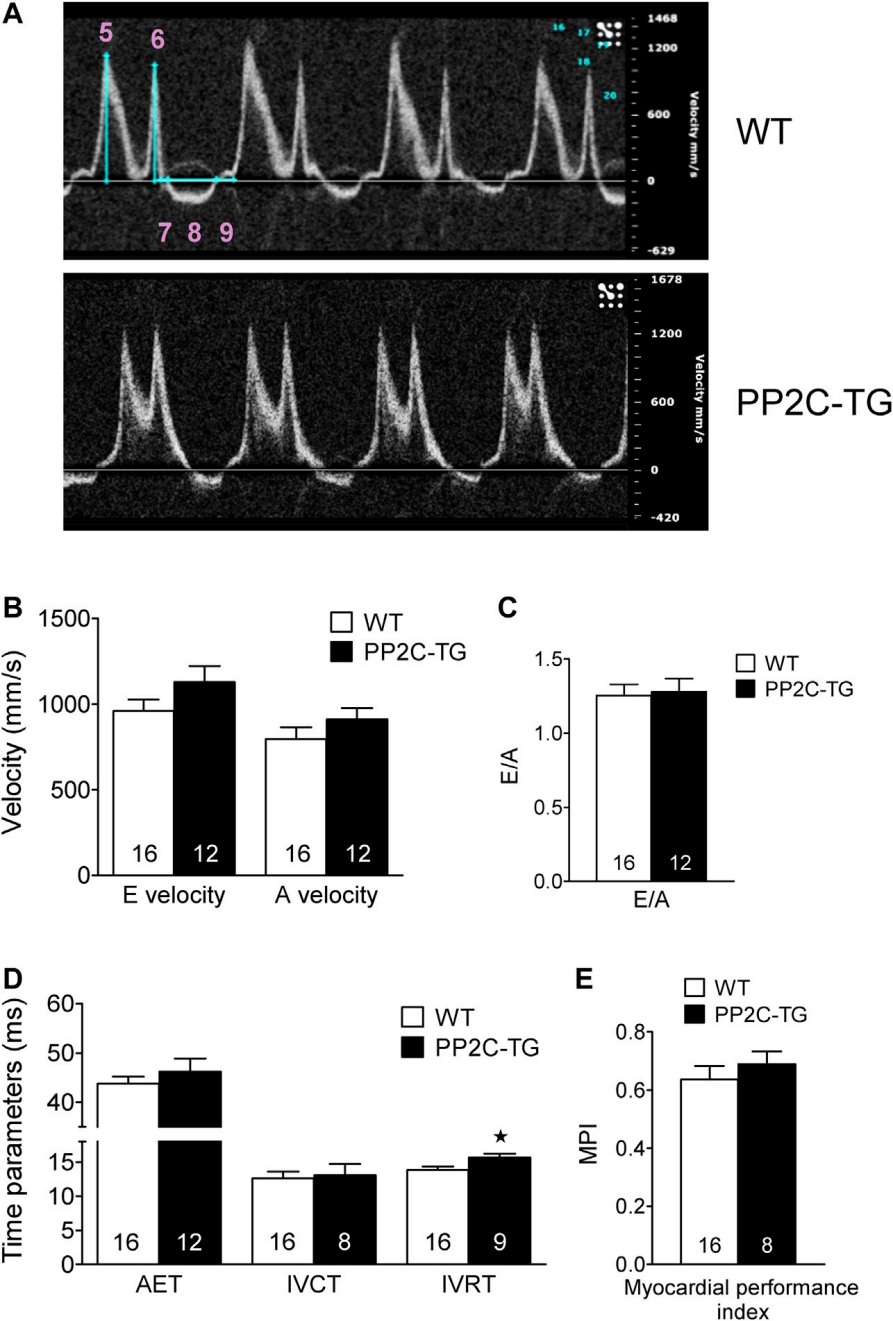
Pulsed wave Doppler echocardiography. **(A)** Mitral valve, original recordings. 5: Peak flow velocity of the early rapid filling wave (E velocity), 6: Peak flow velocity of the late filling wave due to atrial contraction (A velocity), 7: Isovolumetric contraction time (IVCT), 8: Aortic ejection time (AET), 9: Isovolumetric relaxation time (IVRT). **(B)** E and A velocities in mm/s, **(C)** E/A ratio (dimensionless), **(D)** Time parameters AET, IVCT and IVRT in ms, **(E)** Myocardial performance index (MPI = (IVCT+IVRT)/AET) (dimensionless). Whereas the flow velocities E and A as well as AET and IVCT did not diverge, the IVRT was elevated in PP2C-TG. The MPI (= Tei index) was not affected. ★*p* < 0.05 vs. WT.

**FIGURE 5:**
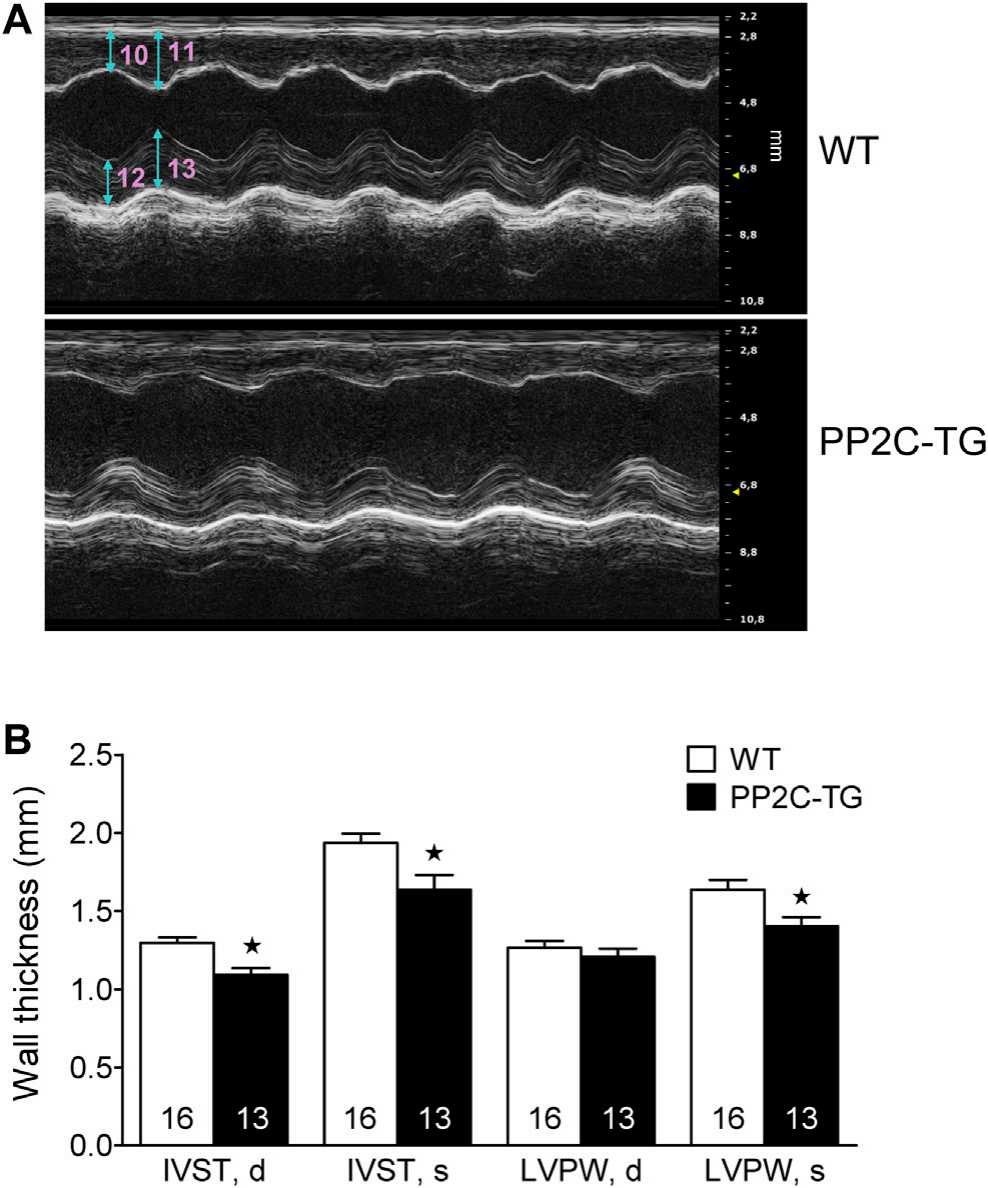
Cardiac dimensions. **(A)** Wall and septum thickness, original recordings. 10: Diastolic Intraventricular Septum Thickness (IVST, d), 11: Systolic Intraventricular Septum Thickness (IVST, s), 12: Diastolic Left Ventricle Posterior Wall Thickness (LVPW, d), 13: Systolic Left Ventricle Posterior Wall Thickness (LVPW, s). **(B)** IVST, d and IVST, s were decreased in PP2C-TG vs. WT. Additionally, LVPW, s was diminished in PP2C-TG compared to WT. ★*p* < 0.05 vs. WT.

**FIGURE 6:**
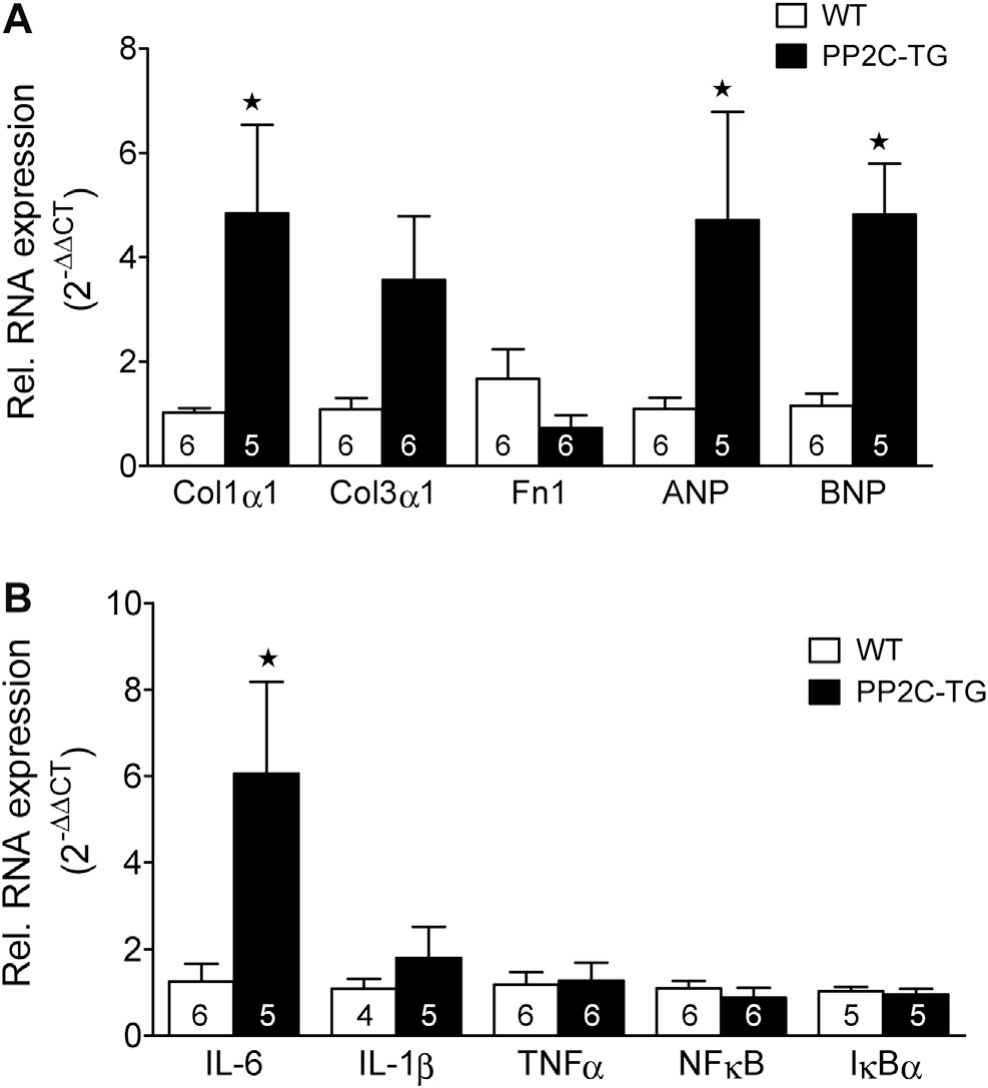
Real time quantitative RT-PCR. **(A)** Markers of fibrosis and hypertrophy. Collagen 1α1 (Col1α1) as a fibrosis marker was elevated in PP2C-TG compared to WT (1.02 ± 0.09 (PP2C-TG) vs. 4.84 ± 1.69 resp.; n = 5–6, *p* < 0.05), whereas Col3α1 evinced a hesitant increase (1.08 ± 0.21 (WT) vs. 3.57 ± 1.22 (PP2C-TG); n = 5–6, *p* = 0.0739), and fibronectin 1 (Fn1) remained unchanged. In PP2C-TG mice, brain natriuretic peptide (BNP) (1.15 ± 0.23 (WT) vs. 4.21 ± 1.0 (PP2C-TG); n = 5–6, *p* < 0.05), was increased, atrial natriuretic peptide (ANP) (1.09 ± 0.21 (WT) vs. 4.71 ± 2.08 (PP2C-TG); n = 5–6, *p* = 0.0876) just hesitantly. **(B)** Markers of inflammation. Interleukin 6 (IL-6) was elevated in PP2C-TG indicative of inflammation (1.25 ± 0.41 (WT) vs. 6.07 ± 2.11 (PP2C-TG); n = 5–6, *p* < 0.05), whereas IL-1β, tumor necrosis factor α (TNFα), nuclear factor κB (NFκB), and inhibitor of κB (IκBα) remained unaltered. Final qPCR data were calculated by the 2^−ΔΔCT^ method (Livak and Schmittgen, 2001). ★*p* < 0.05 vs. WT.

Cardiac overexpression of PP2Cβ was successful in transgenic mice and led to increased levels of PP2Cβ in Western blots as depicted in Figure 1B and quantified in Figure 1D. The Mn^2+^-dependent and therefore putative PP2C phosphatase activity was increased in PP2C-TG heart homogenates (Figure 1B). The protein expression levels of Calsequestrin (CSQ), SERCA, phospholamban (PLB) and the inhibitory subunit of troponin (TnI) were unchanged between PP2C-TG and WT (Figure 1D). Moreover, on membrane discs, PP2Cβ was also able to dephosphorylate [^32^P]-labelled peptides derived from L-type calcium channels (LTCCa1 S1487P) by 30%, MBP-C S282P by 22%, troponin inhibitor S23P by 17%, PLB S16P by 22 ± 11%, B2AR S346P by 30%, PDE3B S10P by 10% and PDE4D3 S129P by 22% under our experimental conditions (n = 3). The overexpression of PP2Cβ led to altered cardiac function in echocardiographic measurements of anaesthetized mice. Under basal conditions, the systolic function of the left ventricle was impaired, as assessed from measuring the left ventricular ejection fraction (EF, or fractional shortening, Figure 2). Stimulation of cardiac β-adrenoceptors by intraperitoneal injection of isoproterenol increased EF (and fractional shortening and heart rate) in WT and PP2C-TG but to a lesser extent in PP2C-TG compared to WT. Under basal conditions (no drug addition), the beating was not different in PP2C-TG and WT (Figure 2). However, to assess right cardiac function, the flow through the vena pulmonalis was studied by means of Doppler ultrasound but no differences were noted between WT and PP2C-TG (Figure 3). Here, only diastolic velocity was decreased in PP2C-TG compared with WT (Figure 3B). However, there were signs of dilatation indicative of a dilatory cardiomyopathy (Figure 3). Moreover, differences in EF between PP2C-TG and WT remained after β-adrenergic stimulation (by intraperitoneal injection of isoproterenol and echocardiography): isoproterenol in PP2C-TG and WT increased EF but to a lesser extent in PP2C-TG compared with WT (Figure 2). In addition, the injection of isoproterenol increased heart rate less in PP2C-TG than WT. Because this concerns the flow through the mitral valve and, thus, left cardiac diastolic function, the E waves and A waves were assessed (Figure 4). Because of the heart rate dependency of the E/A ratio, the heart rate at the moment of the measurement was calculated and found to be comparable between WT and PP2C-TG mice (WT: 522.6 ± 8.8 bpm; PP2C-TG: 518.3 ± 12.8 bpm; n = 12–16). Under these conditions, the isovolumetric relaxation time (IVRT) was prolonged in PP2C-TG compared with WT. As another functional parameter of left ventricular function, the systolic and diastolic interventricular septal thicknesses were smaller in PP2C-TG compared with WT (Figure 5). Likewise, the systolic left ventricular posterior wall thickness was lower in PP2C-TG compared with WT. As classical parameters of heart failure, the mRNA expression of natriuretic peptides (brain natriuretic peptide, BNP and atrial natriuretic peptide, ANP) in PP2C-TG was much larger than in WT (Figure 6A). IL-6 mRNA was increased in PP2C-TG related to WT (Figure 6B), whereas other parameters of inflammation were not significantly different between PP2C-TG and WT (Figure 6B).

**FIGURE 7:**
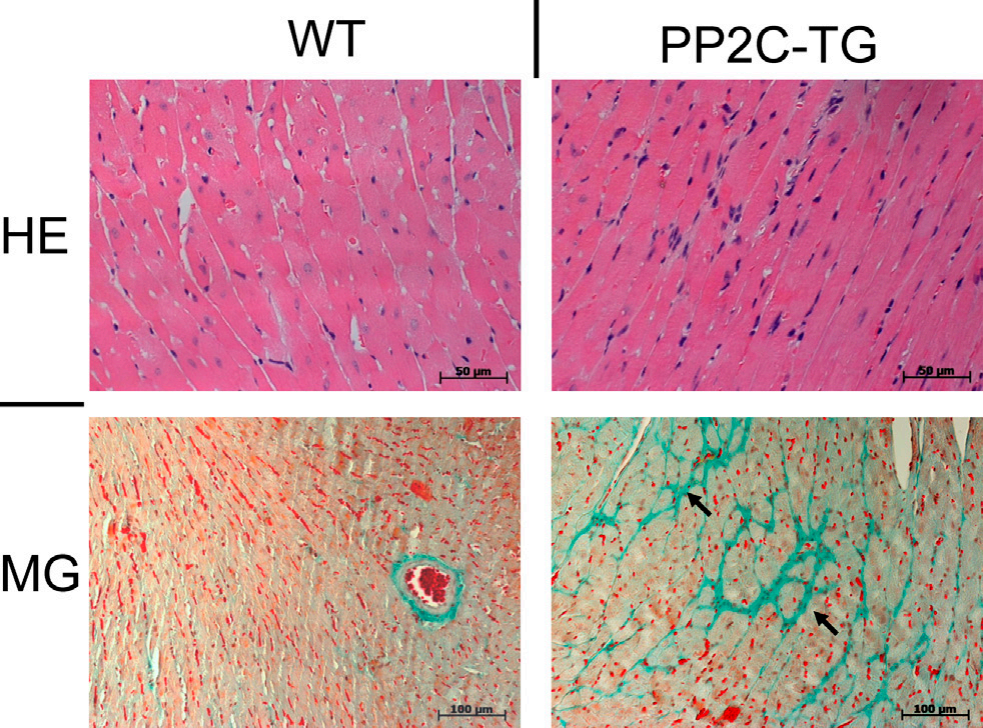
Histology. By hematoxylin/eosin (HE) staining no difference in ventricular tissue structure was detected. Masson Goldner (MG) staining: focal fibrosis (arrows) was found in PP2C-TG ventricular tissue.

In the histological studies, evidence was found for increased fibrosis in PP2C-TG compared with WT (Figure 7), while the hematoxylin/eosin staining results were not altered (Figure 7).

**FIGURE 8:**
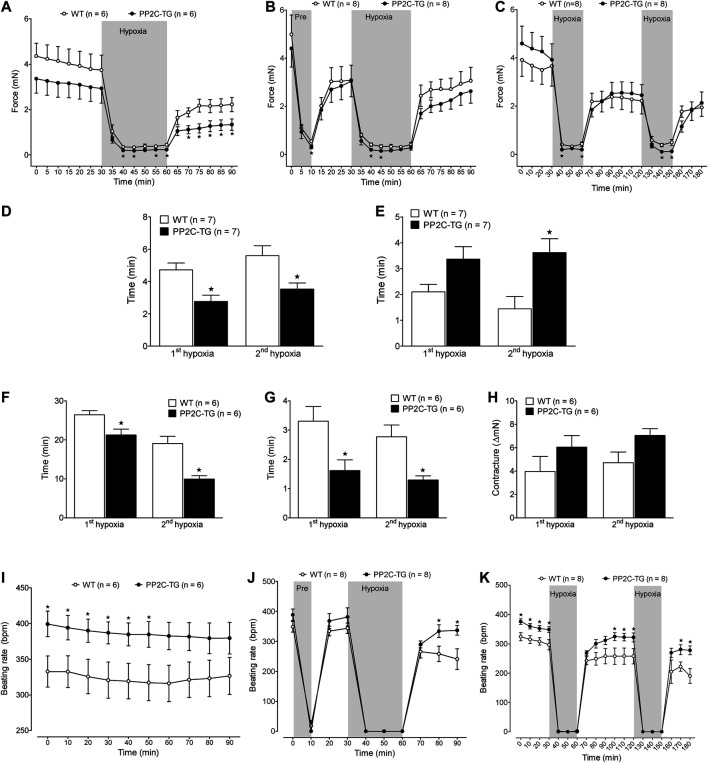
Isolated atrial preparations. **(A–C)** Force of contraction of isolated electrically driven (1 Hz) left atria from PP2C-TG and WT mice demonstrate the experimental protocols. Under basal conditions, contractility was not different between PP2C-TG and WT atria but during hypoxia, left atria of WT mice showed a more pronounced contractility compared to PP2C-TG littermates. **(A)** 30 min hypoxia, **(B)** 10 min hypoxia for preconditioning followed by 30 min hypoxia, **(C)** repeated hypoxia, 30 min each. **(D)** Under hypoxic conditions, force decline (time to 50% of basal force) was faster in PP2C-TG left atria than in WT. **(E)** After reoxygenation, recovery time (time to 50% of maximum force after reoxygenation) was prolonged in PP2C-TG left atria compared to WT. **(F)** During hypoxia, left atrial preparations lose their ability to completely relax. PP2C-TG atria developed contractures earlier than WT atria. **(G)** In contrast, during reoxygenation contractures receded faster in PP2C-TG atria compared to WT. **(H)** Maximum contracture was more pronounced in PP2C-TG atria than in WT atria. **(I–K)** Beating rates (bpm) of isolated spontaneously beating right atria were higher in PP2C-TG than in WT under basal conditions **(I)** as well as at the end of the recovery phase after surviving hypoxia **(J,K)**. ★*p* < 0.05 vs. WT.

Under conditions of hypoxia, force in the left atrium fell faster in PP2C-TG compared with WT (Figure 8A). Moreover, in preconditioning, these differences persisted (Figure 8B). Also, in repeated similar times of hypoxia, the force declined faster in PP2C-TG compared with WT (Figure 8C). These differences were more evident when we plotted time to 50% of force decline (Figure 8D). During hypoxia, left atrial preparations lose their ability to completely relax and developed contractures. Likewise, the differences between PP2C-TG and WT could be noted when we measured the time to 50% increase in diastolic tension (Figure 8F). PP2C-TG atria developed contractures faster than WT atria. In contrast, during reoxygenation contractures receded faster in PP2C-TG atria compared to WT (Figure 8G). The analyses of the before mentioned parameters for a single hypoxia were similar to the first hypoxia in the repeated hypoxia studies (Figures 8D–H) and hence are not shown. Moreover, also the preconditioning data were similar to the repeated hypoxia studies and are not shown separately. Interestingly and unexpectedly, the basal beating rate was higher in PP2C-TG compared with WT (Figure 8I). Using the same protocol as for left atria, the spontaneous beating rate declined in right atrial preparations of PP2C-TG and WT. However, after reoxygenation, the beating rate went up to higher values in PP2C-TG compared with WT (Figure 8J). This pattern remained even after repeated hypoxia (Figure 8K). For comparison, hypoxia was also studied in isolated perfused hearts according to the Langendorff methodology to get data about the ventricular function. Here, a period of 20 min of hypoxia, achieved by stop of perfusion, did not affect the ventricular force generated after reperfusion in both WT and PP2C-TG. Moreover, isoproterenol-induced increase of force and beating rate was unaffected by hypoxia und not different between WT and PP2C-TG. It is noteworthy that the basal beating rate and the beating rate after reperfusion, like in isolated right atria, was higher in PP2C-TG compared to WT (Tables 1, 2).

**TABLE 1:** Basal contractile parameters of isolated perfused hearts of PP2C-TG and WT mice (n = 9, each).

	WT (n = 9)	PP2C-TG (n = 9)
Force of contraction (mN)	13.3 ± 0.91	12.0 ± 0.81
Heart rate (bpm)	384 ± 13.6	466 ± 12.4*
dF/dt max (mN/s)	446 ± 27.0	541 ± 66.8
dF/dt min (mN/s)	−383 ± 50.1	-374 ± 49.3

dF/dt max, min, maximum and minimum of first derivative of force of contraction.

*p < 0.05 vs. WT.

**TABLE 2:** Influence of hypoxia and β-adrenergic stimulation on the contractility of isolated perfused hearts of PP2C-TG and WT mice.

	Normoxia						Hypoxia					
	WT (n = 4)			PP2C-TG (n = 4)			WT (n = 5)			PP2C-TG (n = 5)		
	Basal	Ctr	Iso	Basal	Ctr	Iso	Basal	Hyp	Iso	Basal	Hyp	Iso
Force of contraction (mN)	14.4 ± 1.7	15.7 ± 2.3	19.4 ± 2^#^	12.9 ± 1.5	13.6 ± 0.1	22.6 ± 1^#^	12.4 ± 0.9	12.6 ± 2	17.4 ± 2.2^#^	11.3 ± 0.9	12.4 ± 1.2	18.5 ± 1.7^#^
Heart rate (bpm)	405 ± 11	405 ± 8	560 ± 19^#^	450 ± 9	438 ± 10	560 ± 18^#^	369 ± 21	370 ± 16	537 ± 36^#^	479 ± 20*	452 ± 26*	592 ± 25^#^
dF/dt max (mN/s)	461 ± 43	553 ± 44	814 ± 91^#^	541 ± 67	544 ± 26	1,071 ± 18^#^	435 ± 38	436 ± 58	762 ± 99^#^	484 ± 33	494 ± 34	870 ± 106^#^
dF/dt min (mN/s)	−430 ± 104	−535 ± 107	−941 ± 26^#^	−374 ± 49	−406 ± 37	−978 ± 60^#^	−346 ± 42	−454 ± 65	−808 ± 106^#^	−424 ± 74	−413 ± 57	−829 ± 145^#^

Ctr, time matched to hypoxia but under normoxic conditions; Hyp, hypoxia by stop of flow followed by reperfusion; Iso, isoproterenol (1 µM) application at the end of reperfusion; dF/dt max, min, maximum and minimum of first derivative of force of contraction.

*p < 0.05 vs. WT

^#^p < 0.05 vs. corresponding pre-isoproterenol value.

**FIGURE 9:**
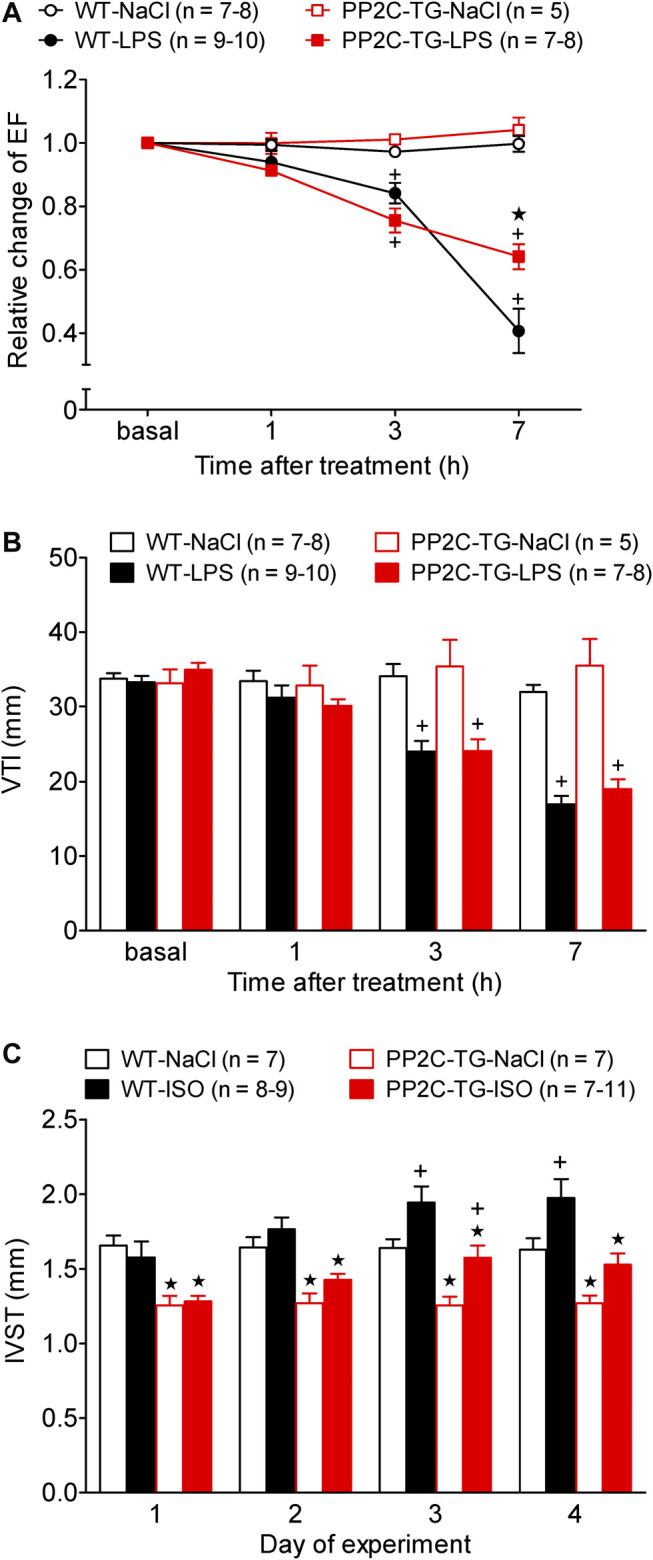
Cardiovascular stress by lipopolysaccharide (LPS) or β-adrenergic stimulation. Cardiac function was determined by echocardiography under basal conditions and after stress induction. **(A)** Time dependent effect of LPS or NaCl as control on left ventricular ejection fraction (EF) in WT and PP2C-TG. Ordinate: relative change in ejection fraction (pre LPS values = 1). Abscissa: hours after LPS injection. **(B)** Time dependent effect of LPS or NaCl on the flow through the aorta measured as velocity time integral (VTI) by Pulsed wave Doppler echocardiography of WT or PP2C-TG. **(C)** Intraventricular septum thickness (IVST) of WT or PP2C-TG. Mice were injected intraperitoneal with isoproterenol (ISO) or NaCl as control and always 5 min thereafter echocardiography was performed. Ordinate: IVST in mm. Abscissa: days of drug treatment. ★*p* < 0.05 vs. WT; ^+^ *p* < 0.05 vs. NaCl.

Moreover, left ventricular function can be impaired in many mammals by injecting LPS. Here, a putative cardioprotective role of PP2C could become apparent: while LPS impaired EF in PP2C-TG and WT, the percentile decrease in EF was more pronounced in WT than in PP2C-TG (Figure 9A). In contrast, the flow through the aorta was reduced after LPS treatment in both PP2C-TG and WT to the same extent, here shown as reduced velocity time integral (VTI, Figure 9B). Cardiac function could be impaired by repeated injection of isoproterenol (Figure 9C). Here, in a different set of animals than those in Figure 5, the same decline in septal thickness in PP2C-TG vs. WT was noted (Figure 9C). After repeated injections of isoproterenol, which might be regarded as chronic adrenergic activation, less of an increase in septal thickness was noted in PP2C-TG compared with WT (Figure 9C).

**FIGURE 10:**
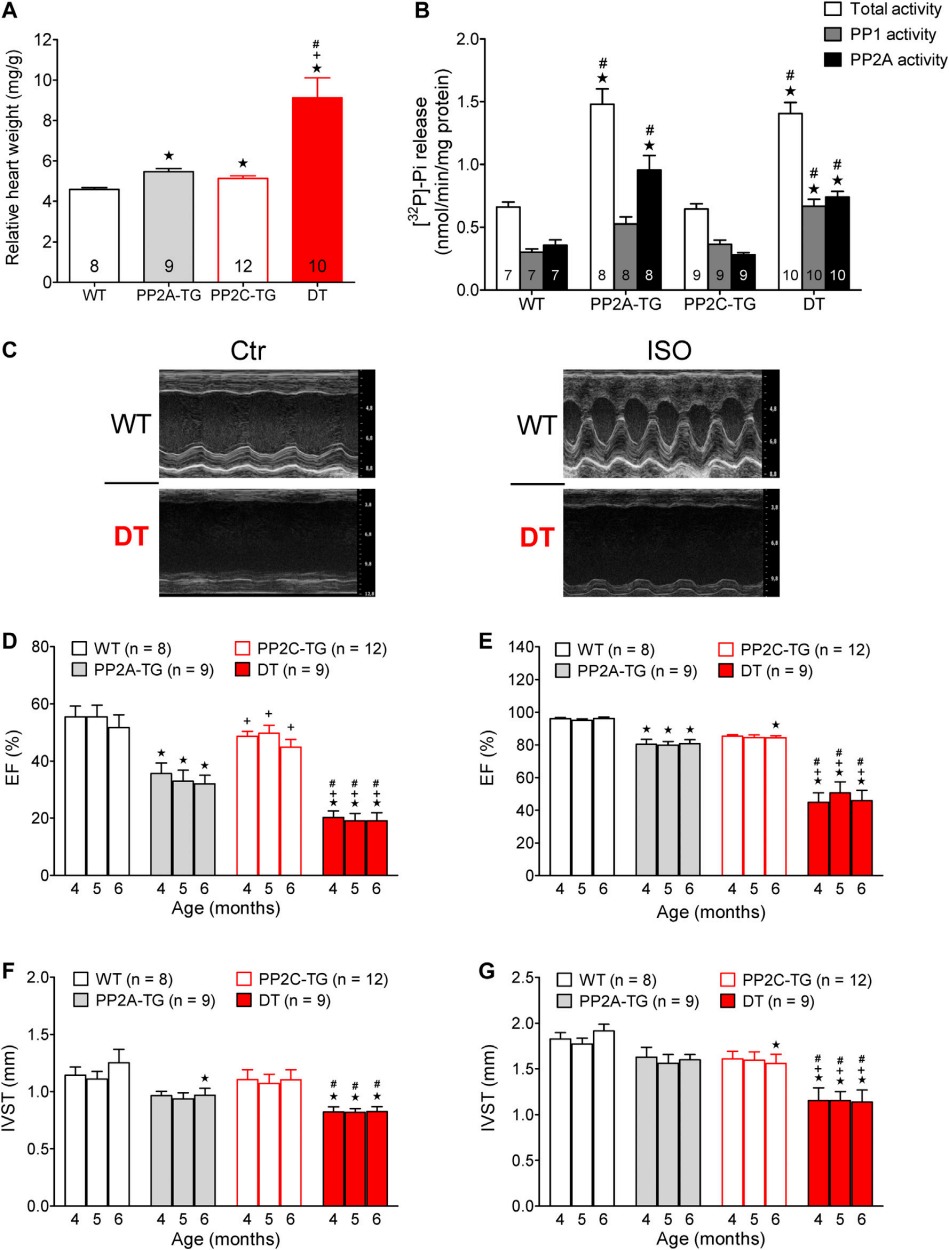
PP2AxPP2C double transgenic mice. **(A)** The relative heart weight of single transgenic mice (PP2A-TG, PP2C-TG) was increased compared to wild type (WT) mice. This effect was much more pronounced in double transgenic (DT) mice. **(B)** Protein phosphatase 1 and 2A activity measured as phosphate release from [^32^P]-labeled phosphorylase a. Separation of PP1 and PP2A activity was achieved by the use of 3 nM okadaic acid. **(C)** M-Mode parasternal long axis view: under basal conditions (Ctr) and after injection of isoproterenol (ISO) in WT and DT. **(D,E)** The ejection fraction is diminished under basal conditions **(D)** and after β-adrenergic stimulation by injection of isoproterenol **(E)** especially in DT mice. **(F,G)** The systolic interventricular septum thickness (IVST) was decreased in DT under basal **(F)** and stress **(G)** conditions. ★*p* < 0.05 vs WT, ^+^ *p* < 0.05 vs PP2A-TG, ^#^ *p* < 0.05 vs. PP2C-TG, numbers of animals are given in columns or brackets.

Furthermore, it was of interest to measure the age-dependent decline in EF in PP2C-TG compared with WT. In addition, we had reported before on an age-dependent worsening of cardiac function accompanied by cardiac hypertrophy in PP2Ac-overexpression mice, which were studied for comparison. Moreover, we wanted to test the hypothesis that the detrimental effects of cardiac overexpression that both PP2A and PP2C might add or even potentiate. Hence, we crossbred PP2A-TG with PP2C-TG: this gave us the unique opportunity to follow the age-dependent decline in left ventricular function (judged by echocardiography and at the end by gravimetry: Figure 10) in monotransgenic and double transgenic (DT) mice. Therefore, we measured left ventricular EF with echocardiography in anaesthetized mice, namely at four, five and six months of age. EF was smaller in PP2A-TG than in PP2C-TG, and this difference compared with WT was apparent after four to six month. DT mice fared much worse than WT, PP2A-TG or PP2C-TG mice (Figure 10). Likewise, the EF was smaller under basal conditions in DT (Figure 10D) and also after isoproterenol (Figure 10E) than in the other genotypes at the beginning. Moreover, the systolic thickness of the interventricular septum was reduced in DT (Figure 10F). The relative heart weights were found to be mildly elevated in PP2A-TG and PP2C-TG but greatly enhanced in DT (Figure 10A). Phosphatase 1 activity amounted to 0.3015 ± 0.026 nmol/mg protein/min in WT, 0.886 ± 0.124 nmol/mg protein/min in PP2A-TG, 0.3635 ± 0.0351 nmol/mg protein/min PP2C-TG and 0.6659 ± 0.0566 nmol/mg protein/min in DT, respectively. On the other hand, PP2A activity was 0.3587 ± 0.0422 nmol/mg protein/min in WT, 0.5719 ± 0.067 nmol/mg protein/min in PP2A-TG, 0.2807 ± 0.0167 nmol/mg protein/min PP2C-TG and 0.740 ± 0.043 in nmol/mg protein/min in DT, respectively (n = 7–10). This means, PP2A (but also PP1) activity is elevated in PP2A-TG and DT vs. WT (*p* < 0.05). The protein phosphatase activity data are visualized in Figure 10B.

## Discussion

**FIGURE 11:**
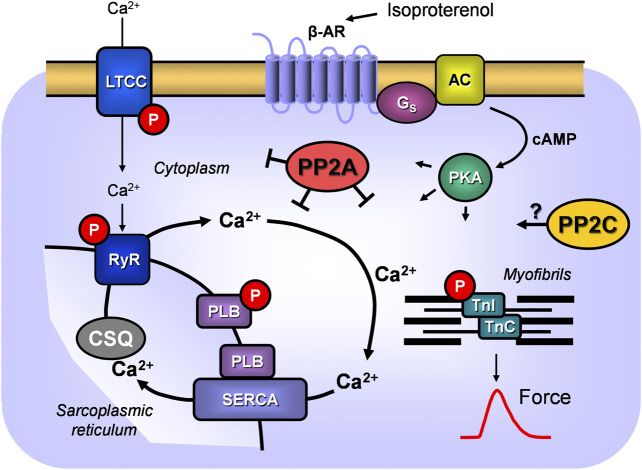
Schematic illustration of signal transduction via β-adrenoceptors in cardiomyocytes and the role of PPs. β-adrenoceptor (β-AR) stimulation by isoproterenol leads via stimulatory G-proteins (G_s_) to increased activity of adenylyl cyclase (AC) which increases cAMP in the cytosol and activation of a cAMP-dependent protein kinase (PKA). This kinase can phosphorylate (P) many cardiac proteins: here phosphorylation of the L-type Ca^2+^channels (LTCC) with subsequent increase of Ca^2+^ flow through the sarcolemma, phosphorylation of the ryanodine receptor 2 (RYR) of the junctional sarcoplasmic reticulum or phosphorylation of phospholamban (PLB) which increases the activity of the sarcoplasmic reticulum Ca^2+^ ATPase (SERCA) and thereby Ca^2+^ uptake into the sarcoplasmic reticulum where Ca^2+^ is stored by calsequestrin (CSQ). Moreover, phosphorylation of the troponin inhibitor (TnI) is indicated which enhances relaxation of the heart. These phosphorylations can be reversed by PP2A and/or PP2C.

A new finding of the current study is the first successful overexpression of PP2Cβ in the living mammalian heart. This model was used to assess the presumptive role of PP2Cβ under basal conditions (Figure 11). In order to facilitate comparison of our findings with the biochemical findings of the working group of S. Klumpp, who provided the PP2C cDNA, we decided to use the bovine PP2C to generate transgenic mice (e.g., Klumpp et al., 1998; Selke et al., 1998). Because PP2Cβ may play a larger role in cardiac disease after appropriate stimuli, transgenic animals or their cardiac preparations were studied under stressful conditions. We noticed differences between transgenic and WT cardiac preparations or animals that might be clinically relevant or warrant further studies.

Surprisingly, the spontaneous heart rate in isolated right atrial preparations and in isolated perfused hearts was higher in PP2C-TG than WT. In contrast, there was no difference under basal conditions in the anaesthetized PP2C-TG and WT animals using echocardiography. One could reconcile these results by suggesting that the vegetative nerve system *in vivo* reduced the intrinsically increased heart rate *in vitro*. The higher basal activity in the isolated right atrium and isolated heart might be because of dephosphorylation of phosphodiesterase (PDE) 3: the dephosphorylation of PDE is known to inactivate PDE3. Likewise, PP2C can dephosphorylate the β_2_-adrenoceptor, increasing its activity: both events would lead to higher cAMP levels in the sinus node, more activation of HNC4 and, thus, higher beating rates. However, this is hypothetical and requires further study.

Our histological data indicate fibrosis in PP2C-TG. These data are plausible and might partially explain the problems that the hearts had when trying to relax. This impairment of relaxation can be clearly seen in intact animals in the enhanced time in IVRT, an accepted parameter for cardiac relaxation (Kotecha et al., 2017).

In hypoxia in intact rats, the expression of PP2C on protein level is increased in the heart (Flores et al., 2020). PP2C can dephosphorylate a kinase called AMPK, and this can lead to cardiac hypertrophy (Sung et al., 2015). Isoforms of PP2C have been localized to the cardiac mitochondria and have been claimed as important for energy metabolism and, thus, also in hypoxia (Joshi et al., 2007).

In the eukaryotic model organism *Saccharomyces cerevisiae*, a PP2C isoenzyme has been noted to dephosphorylate, thereby regulating stress (in this case hypothermia)-related proteins (Sharmin et al., 2014). At least in yeast, PP2C can also lead to autophagy (Memisoglu et al., 2019), and autophagy can contribute to cardiac failure.

Cardiac stress leads to increased expression of HSP70 (e.g., Meissner et al., 2000; Vahlhaus et al., 2005; review: Nair and Sharma, 2020). HSP 70 can be dephosphorylated by PP2C, and this can alter the location and function of HSP 70 in the mitochondria of mammalian cells (Zemanovic et al., 2018).

The fact that intraperitoneal injected isoproterenol increased the beating rate in the heart in living animals to higher values in WT than in PP2C-TG might indicate that PP2C, at least in PP2C-TG, plays a role in the sinus node of the heart (as hypothesized above for isolated atria). Indeed, some claim that phospholamban phosphorylation in sinus node cells of the rabbit contributes to the regulation of the heart beat (Yaniv et al., 2015). On the other hand, phosphorylated PLB is an excellent substrate for PP2C (based on our present data and MacDougall et al., 1991), suggesting that dephosphorylation of proteins such as PLB might explain alterations in beating rates in PP2C-TG *in vitro* and *in vivo*.

Consistent with hypertrophy and the functional impairment of the PP2C-TG heart, an increased expression of cardiac ANP and BNP was noted: this is an increase in natriuretic peptides that is typically present in human heart failure and in many animal models of genetically induced, drug induced or aortic banding–induced heart failure (for a review, see Rabkin and Tang, 2020) and hence is plausible. LPS treatment is a commonly used stress to impair myocardial performance in living animals, and several transgenic mice have been reported to offer protection against this decline in cardiac performance because of LPS (e.g., Ma et al., 2018). We have described that the overexpression of PP5 in the heart was protective of force generation against LPS treatment (Neumann et al., 2019). Repeated short term (for several days or weeks) isoproterenol application is a classic tool to induce cardiac hypertrophy and has been used in many laboratory animals (for a review, see Colucci, 1998), including rats (e.g., Linck et al., 1998) and mice (e.g., Ma et al., 2018).

A time-dependent cardiomyopathy and fibrosis and decline of left ventricular function in PP2A-TG was noted before by us (Gergs et al., 2004) and could be corroborated here in this new set of mice. Cardiomyopathy and impaired function have been reported for mice overexpressing PP1 (Carr et al., 2002; Pathak et al., 2005; Brüchert et al., 2008), PP2B (Wilkins and Molkentin, 2004) or PP5 (Gergs et al., 2012; Gergs et al., 2019c) and can now be extended to PP2C overexpressing mice. The substrates of PP2C and PP2A are overlapping; hence, it is plausible that hypertrophy is also additive (Figure 11).

Indeed, in a companion study, we noted that co-overexpression of PP2A and PP5 led to an additive time-dependent decline in cardiac function accompanied by an increase in mortality in DT mice (Köpp et al., 2018; Dörner et al., 2019).

Another useful application of PP2C-TG will be in the study of phospho-histidine phosphorylation in the heart. This is an understudied area because phospho-histidine is more liable to hydrolysis than phospho-serine or phospho-threonine and was initially overlooked. However, the phosphorylation of histidine occurs, for instance, in G-proteins (which play an important role in cardiac signal transduction) and is dephosphorylated quite effectively by PP2C (Mäurer et al., 2005). Moreover, many other substrates of PP2C have been described *in vitro* (Wieland et al., 2010), and our PP2C mouse may offer unique possibilities to study these substrates in the beating heart.

Finally, our data are consistent with the role of inflammation with the initiation of heart failure in PP2C-TG and DT: parameters such as IL-6 are higher in PP2C-TG than in WT. One may ask why PP1 activity is increased in PP2A-TG mice and DT. However, this is consistent with our previous work in PP2A-TG mice, where we noted an increase in PP1 activity because of lower expression of I-2, an endogenous inhibitory protein of PP1 and reduced phosphorylation and, thus, less action of inhibitor-1 of PP1 (I-1), which we and others reported before (Neumann et al., 1997; Neumann et al., 1999; Mishra et al., 2002; Gupta et al., 2003; El-Armouche et al., 2004).

Limitations of the study are the missing protein data for hypertrophic and inflammatory pathways. Therefore, we are currently not able to demonstrate a clear correlation between cardiac function and a molecular mechanism. However, this requires further study with larger groups of animals.

In summary, we present initial evidence for a putative role of PP2C in the heart in health and disease. Indeed, PP2C might be a druggable target in the heart.

## Data Availability Statement

The raw data supporting the conclusions of this article will be made available by the authors, without undue reservation.

## Ethics Statement

The animal study was reviewed and approved by animal welfare committee of the University of Halle-Wittenberg, Halle, Germany.

## Author Contributions

Designed the research: JN, UG, Wrote the manuscript: UG, JN, Provided materials: SR, UK, FM, Performed experiments: PAB, PEB, MJ, TB, FW, HW, IB, and JR, Analyzed data: PAB, PEB, MJ, TB, FW, HW, IB, and JR.

## Funding

We acknowledge the financial support within the funding program Open Access Publishing by the German Research Foundation (DFG).

## Conflict of Interest

The authors declare that the research was conducted in the absence of any commercial or financial relationships that could be construed as a potential conflict of interest.
